# The Centre for Evidence-Based Orthopaedics Tool for the Implementation of Evidence-Based Practice

**DOI:** 10.7759/cureus.79835

**Published:** 2025-02-28

**Authors:** Line L Houkjær, Dennis W Hallager, Stig Brorson

**Affiliations:** 1 Centre for Evidence-Based Orthopaedics, Zealand University Hospital, Køge, DNK; 2 Department of Clinical Medicine, University of Copenhagen, Copenhagen, DNK

**Keywords:** clinical implementation, evidence-based medicine, evidence-based practice, evidence practice gap, health behavior, implementation science, implementation tool, medical education, motivation, wrist fracture

## Abstract

Introduction

Updating practices can be challenging in evidence-based practice when integrating the evidence, clinical expertise, and patient values and preferences. Implementing evidence-based practice requires individual, collective, and organizational behavioral changes. Effective behavior change interventions are necessary to facilitate the implementation of new evidence. We propose a tool to facilitate the implementation of evidence in clinical decision-making by changing healthcare providers' behaviors.

Methods

The Centre for Evidence-Based Orthopaedics (CEBO) tool was developed to bridge the gap between evidence and practice in a hospital department. The development process follows a Plan-Do-Study-Act (PDSA) structure and is based on the COM-B model (Capability, Opportunity, Motivation, Behavior) and the Theoretical Domains Framework (TDF), which recognize that effective behavior change necessitates addressing factors at both individual and organizational levels. The CEBO tool consists of four phases, guiding the process from identifying an evidence-practice gap to evaluating behavioral changes following implementation.

Results

We applied the CEBO tool to two surgical cases, which led to substantial behavioral changes in orthopedic surgeons' treatment choices. Our findings indicate that the CEBO tool is feasible and can influence surgeons' behaviors to align more closely with the best available evidence.

Conclusion

The CEBO tool helps align practice with the best available evidence. Although implementing new practices effectively is time-consuming, it seems achievable with the CEBO tool. Substantial behavioral changes were observed among surgeons in both cases.

## Introduction

Evidence-based practice integrates the best available evidence, clinical expertise, and patients’ values and preferences [1]. The translation of the best available evidence into practice depends on awareness, acceptance, and adoption [2], where practitioners become aware of the evidence (awareness) and accept the validity of the research (acceptance) and research findings become the standard of care (adoption). These phases help bridge the gap between evidence and practice [2]. Numerous barriers can hamper each of the phases. The amount of new knowledge is overwhelming, with more than one million articles published in the PubMed database in 2023, and even if clinicians are aware that high-quality evidence supports a change, they might be reluctant to do so anyway [3]. There is a delicate balance between a certain conservatism that protects patients from potentially harmful treatments based on spurious research results and an openness toward new, promising treatments or abandonment of ineffective treatments [4].

Meta-studies presenting evidence to clinical decision-makers, such as guidelines, web-based manuals, and various synopses and review formats, are available. However, the availability of these tools does not ensure their use in clinical practice, and their presence does not automatically lead to adoption in clinical settings [5-8]. This adoption can encounter various barriers, which may require individual, collective behavior, and/or organizational changes. By facilitating changes in clinical practice at the departmental level, implementing evidence-based practice can also influence broader organizational policies.

The COM-B model (Capability, Opportunity, Motivation, Behavior) [9] specifies that, for people to change, they need to be capable of change, be motivated to change, and have the opportunity to change [9]. The Theoretical Domains Framework (TDF) identifies 12 domains essential for behavior change: (1) knowledge, (2) skills, (3) social/professional role and identity, (4) beliefs about capabilities, (5) beliefs about consequences, (6) motivation and goals, (7) memory, attention and decision processes, (8) environmental context and resources, (9) social influences, (10) emotion regulation, (11) behavioral regulation, and (12) nature of the behavior [10]. These domains were later validated using the “sort task” validation, resulting in an updated version, where the "nature of the behaviors" was removed and "optimism," "reinforcement," and "intentions" were added, resulting in 14 domains [11]. The domains can be used to identify and understand the barriers and facilitators affecting behavior. The TDF’s 14 domains describe different types of barriers and facilitators that might be encountered [9-12].

Evidence-based surgery involves unique challenges when implementing evidence. Surgical settings are complex, characterized by multidisciplinary teams, hierarchical dynamics, rapidly evolving technologies, and varying levels of evidence robustness. Factors such as individual surgeon’s preferences and patient-specific considerations can significantly affect surgical decision-making [13]. Hierarchical structures are common in clinical departments. They influence the social environment, where more experienced senior clinicians supervise and guide the less experienced junior colleagues. This dynamic can both support and hinder the adoption of evidence-based practices. To ensure that practice is based on the best available evidence, it is paramount that implementation includes all stakeholders, such as clinicians at the senior and junior levels.

We aimed to develop a feasible implementation tool that addresses various barriers and facilitators, drawing from existing literature on the COM-B model and TDF. This work describes the Centre for Evidence-Based Orthopaedics (CEBO) tool for implementing evidence-based practice in an orthopedic department.

## Materials and methods

**Figure 1:**
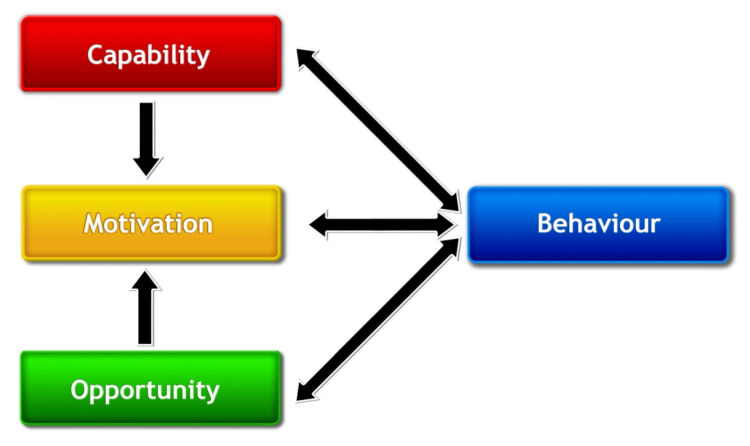
The COM-B model Capability, Motivation, Opportunity - Behavior Model [9]

The CEBO tool was developed to bridge the gap between evidence-based practice and clinical implementation in a hospital department. The development process follows a Plan-Do-Study-Act (PDSA) structure [14] and is based on the COM-B model (Figure 1) [9] and the TDF [11], which recognizes that effective behavior change necessitates addressing factors at both individual and organizational levels.

**Figure 2:**
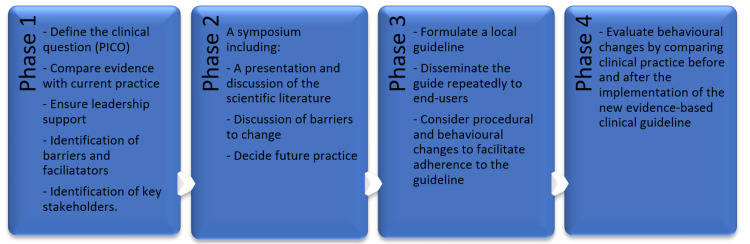
Flowchart illustrating the workflow and the order of the decisions

The CEBO tool includes planning and implementing a change, observing the results, and adjusting for future local interventions. The four phases of the CEBO tool are presented in Figure 2:

Phase 1: the planning

The phase reflects "Plan" in the PDSA structure. 

A project team with clinical and academic competencies is formed. This balance of clinical expertise and academic knowledge is crucial for connecting evidence to practice.

A PICO question [15] defines the scope of the intervention: the patient population (P), the intervention under study (I), the relevant comparator (C), and the benefit and harm outcomes (O). This process ensures that all interventions are anchored in a focused clinical question.

A systematic search is conducted for high-quality guidelines, health technology assessments, reviews, and primary studies, followed by study selection and assessment using established quality assessment tools by the project team.

A review of current practice is conducted using registry data or chart reviews by the project team. Conclusions from relevant evidence are compared to local practice. The comparison of current practice to the best available evidence focuses on identifying the evidence-practice gaps. If the project team identifies an evidence-practice gap, the work continues. The emphasis is on whether guidelines are followed and how local practice differs from evidence, with considerations for specific subgroups. The project team should assess the quality of evidence and related content to address concerns that key stakeholders might raise. These could be major differences in benefits and harms, subgroup variations, or debates regarding outcome measures.

At this point, the head of the department should be involved to ensure leadership support and facilitate the symposium (phase 2). This support is a powerful indicator to end-users about the significance and value of the implementation.

To identify barriers to practice change, the project team maps a typical patient course, setting, and other factors influencing the clinicians’ behavior [16]. According to the COM-B model [9-12], the causal analysis of behavior starts with the question: “What conditions internal to individuals and in their social and physical environment need to be in place for a specified behavioral target to be achieved?” To systematically enhance the identification of all barriers and facilitators, the TDF [6] can be used to discern further and understand the factors influencing facilitators and barriers to change. The domains are elucidated in Table 1 with examples of questions to consider. This process can involve surveys or clinician interviews to assess these behavioral determinants qualitatively.

**Table 1:** TDF in relation to COM-B, along with examples of questions to consider. The TDF consists of 14 domains and can be used to identify and understand the facilitators and barriers to change. COM-B model (Capability, Opportunity, Motivation, Behavior)[9], Theoretical Domains Framework (TDF) [11]

Domain	Examples of questions to consider:
Opportunity	
Environmental context and resources	Barrier: Are there barriers to physical, infrastructural, and available resources that could prevent implementation? Is the environment conducive to change? Facilitator: Are there resources and a supportive environment to enable change?
Social influencers	Barrier: Are there barriers related to social interactions (leaders, colleagues, other staff, or patients)? Facilitator: Are there positive social influences or leaders advocating for the change?
Capability	
Knowledge	Barrier: Are there gaps in knowledge, awareness, or acceptance of evidence? Facilitator: Do the individuals have access to the required information and training?
Skills	Barrier: Are there barriers to skills (physical, technical, or interpersonal)? Facilitator: Do the individuals have the necessary skills and expertise, or is there support for skill development?
Beliefs about capabilities	Barrier: Is there doubt in individuals’ self-confidence, self-efficacy, or competence to make a change? Facilitator: Is there encouragement or mentoring to boost individuals’ self-confidence in their abilities?
Memory, attention, and decision processes	Barrier: Are there barriers in relation to memory, attention, and decision processes, such as remembering the knowledge or making a change or decision? Facilitator: Are there reminders or decision aids to support the change?
Behavioral regulation	Barrier: Are there barriers to behavioural regulation, such as breaking habits or action planning, that could prevent implementation? Facilitator: Are there strategies for establishing new routines and action plans to facilitate the change?
Social/professional role and identity	Barrier: Are there conflicts with social or work settings, such as leadership, organizational commitment, professional identity, or boundaries? Facilitator: Are individuals encouraged to align the change with their professional identity and feel a sense of ownership?
Motivation	
Beliefs about consequence	Barrier: Are there concerns about individuals' perceptions of anticipated outcomes and confidence in achieving results? Facilitator: Are there positive expectations about the benefits of the change and confidence in its success?
Reinforcements	Barrier: Is there a lack of feedback (from patients, colleagues, etc.) and, thereby, reinforcement, which could prevent implementation? Facilitator: Does ongoing feedback and positive reinforcement support the change?
Intentions	Barrier: Are there conflicting motivations (money, time, or outcome)? Facilitator: Are there clear intentions aligned with the purpose of the change?
Emotion	Barrier: Are there negative emotions (e.g., anxiety, stress) concerning the change? Facilitator: Are there efforts to address emotional reactions and provide emotional support?
Goals	Barrier: Are there unclear or conflicting goals on what an individual or group wants to achieve? Facilitator: Are goals clearly defined, with everyone working toward shared objectives?
Optimism	Barrier: Are there doubts about a positive outcome? Facilitator: Is there an expectation that the change will lead to positive results?

Documents like guidelines and patient information material that must be updated to align with the evidence base and evolving practice should be identified.

Key stakeholders, including end-users, formal and informal opinion leaders, and other social influencers in clinical decision-making, should be identified. If possible, patient representatives should also be included.

Phase 2: the symposium

The phase reflects "Do" in the PDSA structure.

The project team invites all key stakeholders for a symposium. Key stakeholders would include healthcare providers. This will vary depending on the specific change but could consist of junior doctors, senior doctors, supervisors, nurses, pharmacists, therapists, and others. The identified evidence from the systematic search in phase 1 is shared before the symposium, and participants are encouraged to contribute additional sources. This ensures a discussion on a commonly accepted and pre-defined evidence base. The symposium is planned to facilitate attendance; it could be during work hours to alleviate other responsibilities, such as the closure of the outpatient clinic.

Participants briefly present the evidence at the symposium and outline a summary of current practice. A moderator guides the discussion within the scope of the defined clinical question, and key points from the discussion are documented in a written summary. The symposium aims to create an environment conducive to change that overcomes inertia and, through collectively increased knowledge, provides a positive social impact for change (opportunity). The symposium aims to enhance participants' awareness and acceptance of the evidence (capability) through open discussions. Also, clarifying the intentions and goals for practice change will increase motivation and, if possible, obtain consensus.

At the end of the symposium, potential barriers and facilitators are revisited with a collective brainstorm on factors that must be addressed for implementation. Emphasis should be on environmental context and resources, skills, and behavioral regulations (Table 1).

Phase 3: clinical implementation

The phase also reflects the "Do" in the PDSA structure.

Based on the discussion at the symposium, either a local guideline is formulated or existing guidelines are updated. The project team is supplemented with the representation of end users and other key stakeholder groups. The guideline should provide evidence-based conclusions and explain its application to local practice. Furthermore, it should encourage patient involvement and shared decision-making. If needed, other documentation and patient information are updated.

A communication plan should be established and supported by the department chief. Simple dissemination alone does not result in behavior change [17]. For example, the guideline should be published in the local guideline repository and repeatedly disseminated to the end-users by mail and/or presentation at morning meetings. Case presentations might be helpful. Reminders have been shown to affect implementation [18]. These efforts aim to facilitate memory and attention and impose a positive attitude toward the practice change (Table 1).

Necessary procedural changes may now be planned. These could include new medicaments, procedures, or devices, staff education, altering the patient's course of treatment, and/or adjusting schedules to allow for additional time needed to implement the practice change.

Phase 4: the evaluation

The phase reflects the "Study" and "Act" in the PDSA structure.

After a pre-defined timeframe, the clinician’s behavior is re-evaluated and compared to baseline data. Data collection depends on the practice under study, such as procedure codes, use of implants, amount of medication, or number of referrals. Others encourage the use of statistical process control charts [19]. The results are presented to the department to induce reinforcement for individual end-users (motivation, Table 1). This audit and feedback process can effectively lead to behavior change [20]. To maximize its impact, it is important to provide this feedback consistently in both verbal and written formats [17-18,20]. Finally, the following interventions using the CEBO tool in the local context should be adapted to previous experiences.

## Results

We have evaluated the feasibility of the CEBO tool on two occasions within an orthopedic setting:

Case 1: surgical treatment options for wrist fractures in adults

Most dorsally displaced distal radius fractures (wrist fractures) can be surgically treated with either a plate (volar locking plate) or pinning (percutaneous steel-wire pinning). In 2019, all surgeries for wrist fractures in adults were performed using plates at our institution. Plates require an invasive approach and are several folds more expensive, and we considered complications from plate procedures more severe, although rare. We asked the clinical question: In adults with a wrist fracture requiring surgical treatment, does plating or pinning result in the best functional outcome after one year? The project team applied the CEBO tool. In phase 2, all the surgeons were invited to the symposium, and almost all participated. Based on the best available evidence, we agreed that plates were not superior to pinning after one year. The participants recommended pinning as the primary treatment if a satisfactory reduction and stability could be achieved. A new guideline was prepared and shared with all surgeons and the staff in the operating rooms. Pinning was made standard equipment, whereas plates had to be asked for by the surgeon. During the following year, we reduced the use of plates by 56% [21].

Case 2: surgical versus non-surgical treatment for patients above 60 years with wrist fractures

Wrist fractures in older adults are common and can be treated either surgically or non-surgically with a cast and outpatient follow-up. In our institution, 79% of patients above 60 years of age with a wrist fracture were surgically treated in 2019. We asked the clinical question: In older adults with a wrist fracture, which treatment - surgical or non-surgical - yields the best functional outcomes after six months? The project team applied the CEBO tool. In phase 2, all surgeons were invited to the symposium, and formal and informal leaders attended. The participants agreed that the difference in wrist function six months after surgery was not above the minimal clinically important difference compared with non-surgical treatment. A new guideline was developed and shared multiple times during morning rounds, educational sessions, and when relevant cases were presented. It was also incorporated into the introductory program for surgeons. Additionally, smart phrases were created for quick insertion into medical records. In 2021, the rate of surgical procedures was reduced from 79% to 11% in patients aged 60 or above with wrist fractures.

Barriers and facilitators

The barrier analysis was performed for both cases based on the project team’s experience and knowledge of local logistics and hierarchal structures. We identified the most significant barrier as a lack of knowledge regarding the evidence. In additiion, we acknowledged the potential issue of disagreement with the conclusions derived from the evidence presented. Furthermore, we recognized that experienced colleagues could influence less experienced staff, where senior staff members, including nurses, often establish standard practices. This dynamic can result in resistance to new evidence-based practices if senior staff members are not aligned with the proposed changes. We identified the facilitators with the greatest impact as those that simplify the process, including strong leadership support, the use of smart phrases, and the availability of accessible materials.

## Discussion

Changes in clinical practice often encounter barriers among practitioners [8], and in a setting that prioritizes patient management, the effort can falter due to inertia. 

We present the CEBO tool, a practical resource for promoting behavior change in clinical decision-making where there is an evidence-practice gap.

We have piloted the feasibility of the CEBO tools on two occasions in an orthopedic setting.

The tool utilizes a multi-component approach to address barriers to change, as identified using the TDF determinants of behavior. An implementation strategy that includes multiple components can be more effective than one that relies on a single component [7]. The way the tool is used can differ based on the context [8], particularly how local factors influence clinical decisions and the specific clinical issue being addressed. The significance of each domain and any changes that occur will vary depending on the clinical setting [6].

When planning the intervention, the project team draws on their experience and knowledge of local patient pathways, hierarchical structures, and interdisciplinary influences to identify end users, formal and informal leaders, local opinion leaders, and other key stakeholders influencing local decision-making. The team must be formed with enough insight to identify these stakeholders, as including them in the process is the foundation of the tool.

Leadership support should be secured early to ensure the feasibility of the practice change facilitated by the CEBO tool. Political and economic aspects could influence the willingness to allocate resources for the practice change; thus, changes that encourage less resource-consuming practices might have a higher chance of endorsement.

The symposium in phase 2 mainly addresses clinicians' awareness and acceptance of evidence individually and collectively and discusses adapting local practices to align with this evidence. While striving for consensus, this is not always possible without compromise. If consensus cannot be reached, the project team should carefully consider input from all stakeholders, including minority viewpoints, before deciding on future practice. We acknowledge that the potential lack of consensus and reliance on the project team's decision-making may pose a limitation of the tool.

Ensuring a sense of ownership among all department stakeholders is essential for successfully implementing local practice changes. The symposium fosters an environment that encourages shared knowledge, consensus on practice change, and discussions about the factors influencing social dynamics. Through this collective debate, we can positively impact key factors such as capability and motivational factors, including knowledge, beliefs about capabilities, beliefs about consequences, intentions, emotions, goals, and optimism [9-12].

While phase 2 discusses the current evidence, phase 3 focuses on adopting and implementing the conclusions drawn from it. 

Identifying barriers to these conclusions may require procedural changes. These changes can either promote the adoption of evidence-based behavior or complicate older patterns by increasing the associated challenges. The primary goal is to reduce obstacles to the desired behavior while making engaging in the undesired behavior more challenging.

Other implementation strategies are not included in the CEBO tool. The strategies were selected based on what was feasible and appropriate in this context. For instance, educational outreach visits are not included in the CEBO tool but are effective [18]. This could be included in phase 3 if relevant to the specific practice change or in other specialties.

Phase 3 of the CEBO tool regards developing a new local guideline to address the issue of unimplemented evidence despite existing guidelines on the topic. National guidelines, while valuable, often face significant delays in their preparation and implementation, given the time-intensive process of developing consensus based on available evidence. National guidelines do not cover all clinical topics or may not be developed for specific practice areas. By contrast, preparing a new local guideline through the CEBO tool allows the department to develop and evaluate local guidelines, building ownership among staff. The CEBO tool may improve alignment with the guideline and promote behavior change by incorporating the staff’s perspectives, as emphasized earlier in the discussion.

In phase 4, re-evaluating clinician behavior after a predefined timeframe is crucial for assessing the impact of implemented interventions and ensuring sustained adherence to evidence-based practices.

The type of data collected, such as procedure codes, use of implants, medication amounts, or number of referrals, plays a significant role in measuring the implementation and can influence the accuracy of the evaluation.

Audit and feedback mechanisms have shown a modest effect in promoting behavior change among clinicians [18, 20].

There are similarities between this tool and other frameworks, such as the Knowledge to Action framework [22], and uses the TDF like French et al.'s four-step plan for developing theory-informed behavior change interventions [23]. The CEBO tool offers a description of how each step is carried out in a medical department.

Limitations

While using the CEBO tool has been shown to lead to behavior change, other factors may drive changes. For instance, it is unclear if changes would have occurred even without the tool's application.

In both cases, the suggested practice changes were less resource-consuming. It is plausible that the degree of behavioral change might have been less if a more resource-consuming or expensive practice had been warranted, particularly from the perspective of stakeholders such as department leaders or administrators responsible for resource allocation. Testing the implementation tool at the same department where it was developed could positively affect attitudes toward promoting behavioral change and facilitate the implementation process. Future research may assess whether initial behavior changes can be sustained without ongoing reinforcement or periodic reevaluation. The long-term sustainability of the changes made using the CEBO tool remains unanswered.

The CEBO tool has only been tested in an orthopedic setting. Generalizability and validation in other clinical specialties and settings are necessary to confirm its broader applicability.

Achieving both individual and collective behavioral change can vary based on context. Although the CEBO tool systematically identifies barriers, complex interpersonal dynamics and entrenched behaviors may still pose challenges. While the CEBO tool draws on the TDF and COM-B models in a structured, practical manner, validation in terms of feasibility, efficacy, sustainability, and stakeholder engagement needs to be established.

## Conclusions

The CEBO tool was developed as a feasible implementation tool to address various barriers and facilitators in behavior change. Drawing from the existing literature on the COM-B model and TDF, it provides a structured approach to align clinical practice with the best available evidence at the department level through a four-phase process. The tool proved feasible in an orthopedic setting and demonstrated its ability to support behavioral change among clinicians.
